# Ergonomic Recommendations in Ultrasound-Guided Botulinum Neurotoxin Chemodenervation for Spasticity: An International Expert Group Opinion

**DOI:** 10.3390/toxins13040249

**Published:** 2021-03-31

**Authors:** Philippe Lagnau, Alto Lo, Ryan Sandarage, Katharine Alter, Alessandro Picelli, Jorg Wissel, Monica Verduzco-Gutierrez, Areerat Suputtitada, Michael C. Munin, Stefano Carda, Omar Khan, Serdar Koçer, Rajiv Reebye

**Affiliations:** 1 GF Strong Rehabilitation Centre, Vancouver, BC V5Z 2G9, Canada; philippe.lagnau@aphp.fr; 2 Canadian Advances in Neuro-Orthopedics for Spasticity Congress (CANOSC), Kingston, ON K7K 1Z6, Canada; aslo@ualberta.ca (A.L.); ryan.sandarage@alumni.ubc.ca (R.S.); prof.areerat@gmail.com (A.S.); stefano.carda@gmail.com (S.C.); omar.khan@hoteldieushaver.org (O.K.); 3 Division of Physical Medicine and Rehabilitation, University of Alberta, Edmonton, AB T6G 2R3, Canada; 4 Faculty of Medicine, University of British Columbia, Vancouver, BC V6T 1Z4, Canada; 5 Functional and Applied Biomechanics Section, Rehabilitation Medicine, Clinical Center, National Institutes of Health, Bethesda, MD 20892, USA; kalter@cc.nih.gov; 6 Department of Neurosciences, Biomedicine and Movement Sciences, University of Verona, 37134 Verona, Italy; alessandro.picelli@univr.it; 7 Neurological Rehabilitation & Physical Therapy, Department of Neurology with Stroke Unit, Vivantes Hospital Spandau, 13585 Berlin, Germany; joerg.wissel@vivantes.de; 8 Department of Rehabilitation Medicine, Joe-R.-and-Teresa-Lozano Long School of Medicine, UT Health San Antonio, San Antonio, TX 78229, USA; monicaverduzco@hotmail.com; 9 Department of Rehabilitation Medicine, Faculty of Medicine, Chulalongkorn University, and King Chulalongkorn Memorial Hospital, Bangkok 10330, Thailand; 10 Physical Medicine and Rehabilitation School of Medicine, University of Pittsburgh School of Medicine, Pittsburgh, PA 15213, USA; muninmc@upmc.edu; 11 Neuropsychology and Neurorehabilitation Service, Department of Clinical Neuroscience, Lausanne University Hospital (CHUV), 1011 Lausanne, Switzerland; 12 Hotel Dieu Shaver Health and Rehabilitation Centre, St. Catharines, ON L2T 4C2, Canada; 13 Centre de Rééducation Hôpital du Jura, 2900 Porrentruy, Switzerland; serdar.kocer@h-ju.ch; 14 Division of Physical Medicine and Rehabilitation, University of British Columbia, Vancouver, BC V6T 1Z4, Canada

**Keywords:** ergonomics, ultrasound-guided, chemodenervation, botulinum neurotoxin, muscle spasticity

## Abstract

Ultrasound (US)-guided botulinum neurotoxin (BoNT) injections are becoming a mainstay in the treatment of muscle spasticity in upper motor neuron syndromes. As a result, there has been a commensurate increase in US-guided BoNT injection for spasticity training courses. However, many of these courses do not emphasize the importance of ergonomics. This paper aims to highlight the importance of ultrasound ergonomics and presents ergonomic recommendations to optimize US-guided BoNT injection techniques in spasticity management. Expert consensus opinion of 11 physicians (4 different continents; representing 8 countries, with an average of 12.6 years of practice using US guidance for BoNT chemodenervation (range 3 to 22 years)). A search using PubMed, College of Physicians and Surgeons of British Columbia database, EMbase was conducted and found no publications relating the importance of ergonomics in US-guided chemodenervation. Therefore, recommendations and consensus discussions were generated from the distribution of a 20-question survey to a panel of 11 ultrasound experts. All 11 surveyed physicians considered ergonomics to be important in reducing physician injury. There was complete agreement that physician positioning was important; 91% agreement that patient positioning was important; and 82% that ultrasound machine positioning was important. Factors that did not reach our 80% threshold for consensus were further discussed. Four categories were identified as being important when implementing ultrasound ergonomics for BoNT chemodenervation for spasticity; workstation, physician, patient and visual ergonomics. Optimizing ergonomics is paramount when performing US-guided BoNT chemodenervation for spasticity management. This includes proper preparation of the workspace and allowing for sufficient pre-injection time to optimally position both the patient and the physician. Lack of awareness of ergonomics for US-guided BoNT chemodenervation for spasticity may lead to suboptimal patient outcomes, increase work-related injuries, and patient discomfort. We propose key elements for optimal positioning of physicians and patients, as well as the optimal setup of the workspace and provide clinical pearls in visual identification of spastic muscles for chemodenervation.

## 1. Introduction

Ergonomics is defined as the study of human factors affecting the worker [physician], with a focus on observing how people interact with their work environment and adapting the workplace to the worker, their abilities and limitations [1]. Oftentimes, the goal of optimizing ergonomics is to decrease workplace injuries and improve safety while increasing workplace efficiency. When applied to physicians using ultrasound (US) guidance for botulinum neurotoxin (BoNT) injections for spasticity, this consists of analyzing the physical relationships between the physicians, their patients, and their work environment to optimize comfort and treatment outcomes.

Traditionally, the targeting of muscles for chemodenervation using BoNT was guided by a combination of anatomical landmarking, direct muscle stimulation with electrical stimulation, and electromyography. However, US guidance is now becoming an increasingly utilized guidance method for chemodenervation in patients with spasticity and may provide greater anatomic accuracy when compared to traditional methods [2,3,4]. Furthermore, US is a reproducible method for assessing muscle structural architecture which may help guide spasticity treatment in the future [5,6].

As higher doses and more frequent dosing of BoNT are now being proposed in the treatment of spasticity [7,8,9], it is imperative that proper targeting of the affected spastic muscles be considered to decrease the risk of iatrogenic BoNT spread into non-targeted muscles.

The use of US for injection of BoNT may decrease the risk of iatrogenic BoNT spread by improving accuracy in targeting certain muscle groups such as the gastrocnemius muscle [10,11,12] and avoiding injection into the neurovascular bundle [3].

As a result of evidence that US-guided BoNT injections for spasticity may improve treatment outcomes [10,11], there has been a proliferation of international training courses for US-guided BoNT ultrasound courses for spasticity management [13,14,15,16,17].

Due to the challenge of using the two-dimensional image produced by ultrasound imaging to create a mental three-dimensional representation, many of these courses focus on muscle identification under ultrasound through pattern recognition; understanding muscle anatomy and related structures through cadaveric dissection with comparison to computed tomography (CT) or Magnetic resonance imaging (MRI) cross-sectional images; and the physics of ultrasound waves to help optimize the ultrasound image for muscle identification [13,17]. Ergonomic teaching, such as patient and physician positioning and proper transducer/probe grip are usually given limited exposure during these training courses. However, proper ergonomics are important to reduce physician-related injuries and reduce patient discomfort. Furthermore, proper ergonomics may potentially improve treatment outcomes by improving the accuracy of muscle identification under ultrasound and reduction of injection-related errors [18].

This is supported by the findings from the European consensus table on the use of BoNT in adult spasticity suggesting that non-response to injections may be affected by multiple factors such as insufficient drug dosage, inaccurate muscle selection, and development of changes in the targeted muscles (i.e., fibrosis, contracture) as well as by inaccurate injections [19]. While studies have extensively focused on ergonomics in diagnostic ultrasound sonography [20,21,22], there are comparatively fewer studies with procedural ultrasound and a search using PubMed, College of Physicians and Surgeons of British Columbia database, EMbase revealed no publications conveying the importance of ergonomics in US-guided chemodenervation for spasticity.

In this narrative review, we outline the importance of ergonomics for US-guided BoNT injections for spasticity management and the need for further studies to better explore its impact on physician and patient outcomes. Using images, we also discuss common ergonomic errors made and suggest ergonomic improvements.

## 2. Ergonomics: The Reality

To our knowledge, there are no studies examining ergonomics in US-guided chemodenervation for spasticity management and its impact on physicians and patients. However, outside of this application, other medical specialists using diagnostic ultrasound imaging and/or US-guided therapeutic injections have highlighted the importance of enhancing ergonomics before use. Given the increasing use of ultrasound as a guidance method for BoNT injections and the fact that optimal workplace ergonomics have been proven to reduce work-related musculoskeletal (MSK) injuries [20], specific ergonomic recommendations in this field may be beneficial.

MSK pain is common among sonographers. In large-scale studies, Pike et al. and Evans K et al. reported that more than 80% of sonographers develop MSK work-related injuries and continue to work despite their MSK pain [21,22]. The shoulder and the neck are the most commonly affected body areas with MSK pain in sonographers. In a more recent study, a survey conducted on 1234 sonographers confirmed that a high percentage (85.5%) continue to work despite the pain and that the most commonly painful body part is the shoulder [23]. As a result of the increasing awareness of MSK induced disorders in sonographers, the 2003 Industry Standards for the Prevention of Work-Related Musculoskeletal Disorders (WRMSDs) in Sonography were revised in 2016, offering recommendations that may assist in the reduction in WRMSDs in users of sonographic equipment [24]. However, these recommendations reflect an “ideal” workplace. In the survey conducted by C. Scholl et al., 26.8% of sonographers identified that these guidelines could not be followed in situations where patients were unable to cooperate due to limited mobility or critical conditions. These patient factors acted as barriers to applying these industry-standard ergonomic scanning techniques [23].

Application of these guidelines may also be difficult in US-guided chemodenervation. Patients with spasticity often have decreased mobility and present with decreased voluntary muscle movements and have specific posturing of their limbs which makes ideal positioning of limbs for ergonomic injections difficult. This may require an assistant to obtain correct exposure or immobilization of the limb when spasms or excessive nociceptive flexion reflexes occur during needle penetration. In other cases, despite the use of an assistant, proper position of the limb is impossible as the patient may have an MSK contracture related to their upper motor neuron lesion resulting in decreased passive range of motion. In addition, US-guided BoNT chemodenervation shares similar challenges to MSK interventional procedures; these include the use of one hand for probe manipulation and the other hand for injecting while maintaining continuous visualization of the target on the screen. Guidelines have explored these concepts for procedures such as central venous cannulas and joint aspiration/injections [25]. However, for patient-related reasons as described above, these are not necessarily translatable to US-guided BoNT chemodenervation. Thus, there are patient and physician factors that can limit ergonomic positioning during these procedures, resulting in increased WRMSDs.

Although not specifically described in spasticity management, the impact of ergonomics has been discussed in a recent publication regarding nerve blocks used in interventional anesthesia [26]. These publications are of interest concerning spasticity management as nerve blocks are becoming more frequently performed to help select muscles for chemodenervation. In a study of novice anesthesia resident’s behaviour during US-guided nerve block procedures, Sites et al. found that there were ergonomic errors that impacted nerve blocks, and these include an arching torso, use of the non-dominant hand holding the needle, or head turned 45° or greater. It was hypothesized that these positions resulted in unintentional probe movement during the procedures [27]. Poor ergonomics were furthermore associated with increased injector fatigue which had potential negative outcomes such as increased time to perform blocks and possible block failure [27]. Thus, the application of these findings to US-guided chemodenervation suggests that poor ergonomics could potentially lead to suboptimal outcomes.

## 3. Results

**Figure 1:**
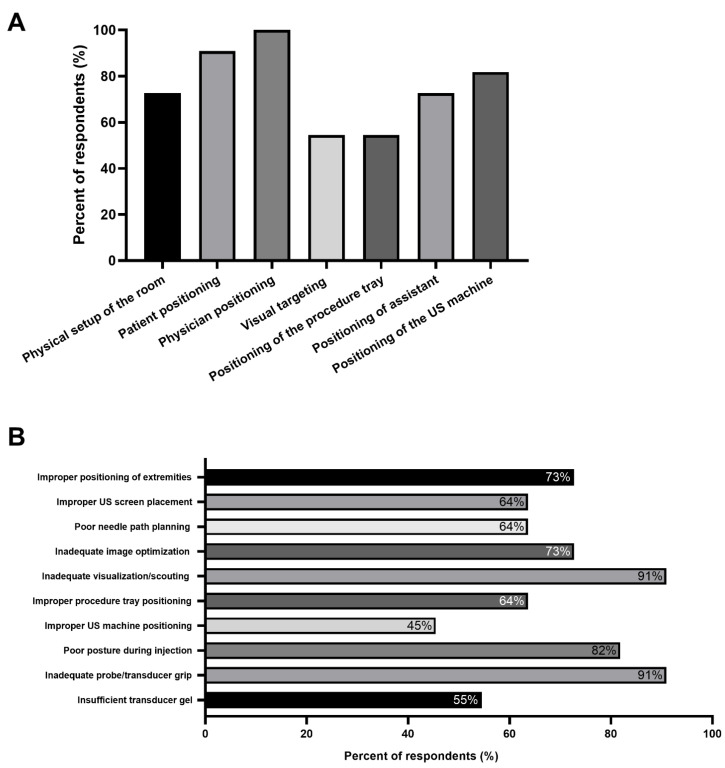
Selected survey responses. (**A**) Elements considered essential to optimize ergonomics for an ultrasound-guided botulinum neurotoxin (BoNT) injection for spasticity management. (**B**) Expert injector observations of common ergonomic errors made by trainees during ultrasound (US)-guided chemodenervation teaching.

There was a response rate of 100% as all 11 injectors replied to the survey. The average years of experience regarding using ultrasound guidance for BoNT chemodenervation among the injectors was 12.6 years (range 3 to22 years) and the average years of experience with teaching medical students, post-graduate training physicians and junior physician staff relating to US-guided BoNT chemodenervation was 10.5 years (range 2 to 20 years; Table 1). Our injectors identified that only 17.3% (range 0 to 50%) of the ultrasound training courses they have attended formally provided training around ergonomics. There was 100% agreement that ergonomics in US-guided BoNT chemodenervation is important in reducing physician injury and 81% agreement that ergonomics is also important for clinics to run efficiently. Concerning ergonomic optimization, 100% of respondents identified that physician positioning was important; 91% identified that patient positioning was important, and 82% identified that ultrasound machine positioning was important. There were variable opinions on whether other ergonomic factors such as being seated when injecting, visual targeting, and other ergonomic suggestions were important considerations during injection (Figure 1A). Consensus opinion regarding common ergonomic errors made by trainees included the following: inadequate grip of transducer (also termed probe) (91%), inadequate visualization/scouting of nearby structures when injecting (91%) and poor posture during injection (82%). Other errors were identified by our injectors, but they did not reach the level of consensus opinion (Figure 1B).

Some questions in the survey did not reach the 80% threshold level to reach a consensus opinion. Discussions were then held between the international experts to elaborate further on the reasons for their responses. The most likely reasons upon discussion were due to the differences in access to resources, space, and staff.

The survey also contained open-ended questions that did not lend themselves well to a written or figurative summary. However, the answers to these open-ended questions were used to develop the international expert group recommendations outlined in the discussion section of this paper.

From these answers and discussions, a consensus opinion on the most common ergonomic mistakes seen by the panel when teaching (Figure 1B), led to four categories identified as being important when implementing ultrasound ergonomics for BoNT chemodenervation for spasticity:

*Workstation Ergonomics:* Room/ultrasound machine set-up.

*Physician Ergonomics:* Optimizing physician position, handling of transducer and needle position.

*Patient Ergonomics:* Optimizing patient position before to ultrasound guided chemodenervation procedure.

*Visual Ergonomics:* Optimizing the ultrasound image for bony landmarks and awareness of pattern recognition for muscle identification, as well as determination of muscle echogenicity.

All 11 experts reviewed and agreed to all of the group recommendations presented in this paper.

## 4. Discussion

### 4.1. Overview

Standards and studies evaluating the proper ergonomics in diagnostic sonography have been previously published. However, none are specific to chemodenervation with BoNT [24,28,29]. In the opinion of our international expert group, optimization of the four categories identified (Workstation ergonomics, Physician ergonomics, Patient ergonomics and Visual ergonomics) may likely improve patient comfort and satisfaction of injection sessions; improve physician comfort and reduce physician injuries; reduce procedure-related errors (such as unintentional probe movement; misidentification of muscles) resulting in better treatment outcomes.

We acknowledge that there may be significant differences in access to resources, space, and staff and the following practical clinical pearls regarding workstation ergonomics, physician ergonomics, patient ergonomics and visual ergonomics are recommendations based on our consensus opinion.

### 4.2. Workstation Ergonomics

#### 4.2.1. Room Dimensions

A room that is 150 square feet in size facilitates maneuverability of machine and equipment around the patient [24]. Equipment cords such as power cables, ethernet cables, and other related cables can either be covered to prevent tripping and facilitate movement of a chair over top of the cables, or neatly positioned in such a way as to not interfere with the injector and assistant movement.

#### 4.2.2. Height Adjustable Table and Comfortable Chair

**Figure 2:**
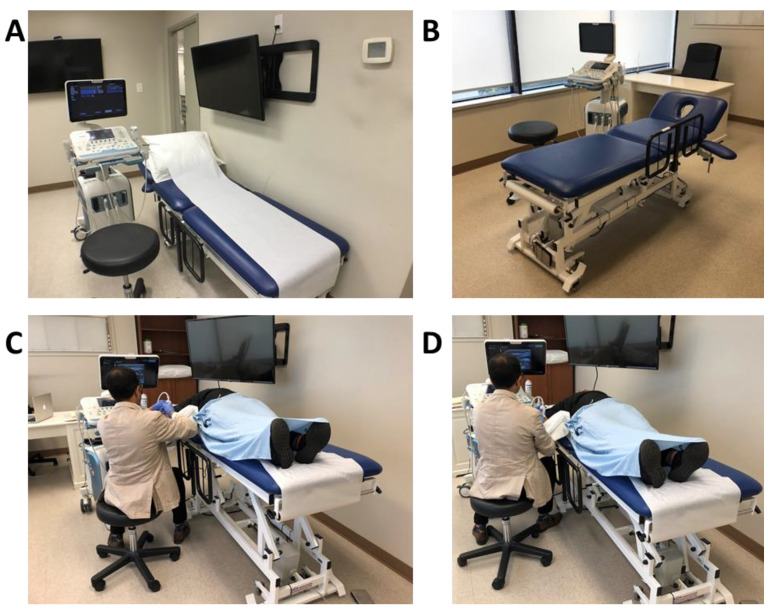
Workstation ergonomics. (**A**) Typical setup of an injection room with the examination table against the wall. In this situation, if a patient requires bilateral injections, it would potentially require the patient to be turned to access the limb closer to the wall. In addition, note the second screen on the wall to help with visualization. (**B**) Ideal workstation setup with examination table centered in the middle of the room with adequate space to move ultrasound machine. (**C**) Suboptimal positioning with a low chair and elevated table result in hiking of the right shoulder and increased strain on the physician’s shoulder. (**D**) Bed height and swivel stool height are at an optimal level to allow for the upper extremity to fall naturally and the shoulder to be relaxed. The position of ultrasound is at eye level, also resulting in a comfortable and neutral position of the cervical spine.

Ideally a height-adjustable examination table can be placed at the center of the room and a swivel rolling chair or examination stool can be used to ease navigation and access to different injection sites (Figure 2A,B) [24]. A comfortable chair and upright position of the body has been shown to reduce the incidence of back pain [30].

#### 4.2.3. Ability to Control Room Lighting, Adjusting Ultrasound Machine Parameters

Adjusting room lighting and ultrasound parameters (Depth, Focus, Gain, Doppler) and selecting the correct choice of transducer frequency (12–17 Hz: superficial muscles/3–5 Hz: deep muscles) are essential to optimize image quality and to ensure precise targeting [31].

#### 4.2.4. Ultrasound Screen at Eye Level

The ultrasound screen should be at eye level on the opposite side of the physician but within arm’s reach to permit ultrasound parameter optimization while scanning before injecting [32]. Ideally, the machine should be directly facing the operator as this results in less rotational movements of the back and neck, decreasing the risk of strain and injury [28]. If equipped with one, the foot pedal should be easily accessible for image acquisition.

#### 4.2.5. Having an Assistant Available

Because both hands are occupied during US-guided injections, ideally, having an assistant present, especially for novice injectors, can facilitate correct positioning of limb and help with dynamic visualization of muscle group by passively moving the muscle while it is being scanned [33].

#### 4.2.6. Small Portable Surface

An easily accessible surface, such as a mobile tray/table should also be within arm’s reach to allow for easy access to additional equipment including alcohol swabs for sterilization, ultrasound gel for scanning, a surface to rest an EMG/nerve conduction machine and towels to help clean up after the completion of the procedure.

### 4.3. Physician Ergonomics

*Neutral Posture:* Maintaining a neutral posture is one of the fundamental principles of proper ergonomics [24]. Certain positions are recognized as optimal in sonography [29]:

(a) Neck should be flexed and not be extended.
(b) Forearm horizontal to ground/examination table.
(c) Arm abduction less than 30°.
(d) Limited radial and ulnar deviation, less than 15° of wrist flexion and extension.
(e) Arm should stay vertical at the side of the body with limited shoulder flexion and extension.

*More specifically with regards to injection under ultrasound guidance:*

**Figure 3:**
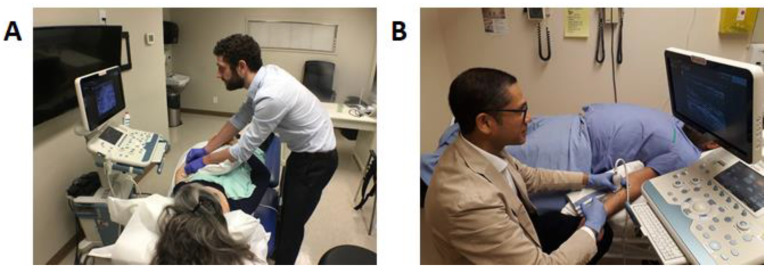
Visual comparison of optimal and sub-optimal ergonomics. (**A**) Sub-optimal ergonomics: The height of the bed is too low for the injector, requiring forward flexion of the trunk. Because of this positioning and because the ultrasound screen is not at eye level, there is a compensatory extension of the neck, resulting in possible neck strain. In addition, both ultrasound machine and targeted limb are away from the injector, resulting in excessive scapular protraction that can result in shoulder pain. (**B**) Correct ergonomic positioning: Notice the injector seated with the ultrasound machine on the same side as the targeted limb so that there is a direct line of sight (i.e., ability to see the patient, the ultrasound screen, and the limb). In addition, note the position of the transducer grip used for an in-plane injection to the left upper limb flexor carpi radialis muscle. The patient is comfortable, with the left arm in optimal positioning for injection. Notice the injector’s neutral wrist position of the right hand with no excessive flexion and needle positioning (needle at 25 degrees insertion and approximately 0.5 cm to 1 cm from the transducer). The ultrasound machine is well-positioned within easy reach as to permit the ability for image optimization.

(f) Attempt to have an ultrasound screen in the same line of sight as muscle to inject to minimize the degree of neck movement and facilitate a smooth transition of gaze from the screen to needle (“look down the barrel”).
(g) Avoid excessive scapular protraction when injecting.
(h) Physicians should be on the same side as the injected limb to avoid reaching over the patient, as well as to avoid excessive scapular protraction from excessive reaching. Figure 3A shows incorrect physician positioning for injection of the upper limb and Figure 3B demonstrates the correct ergonomic position for an upper limb ultrasound guided chemodenervation for BoNT injection.

Seated injections and limiting trunk and neck/flexion: When possible, the physician should be seated and limit trunk and neck/flexion as this facilitates neutral posture [28] and improves stability during an injection (Figure 3B).

**Figure 4:**
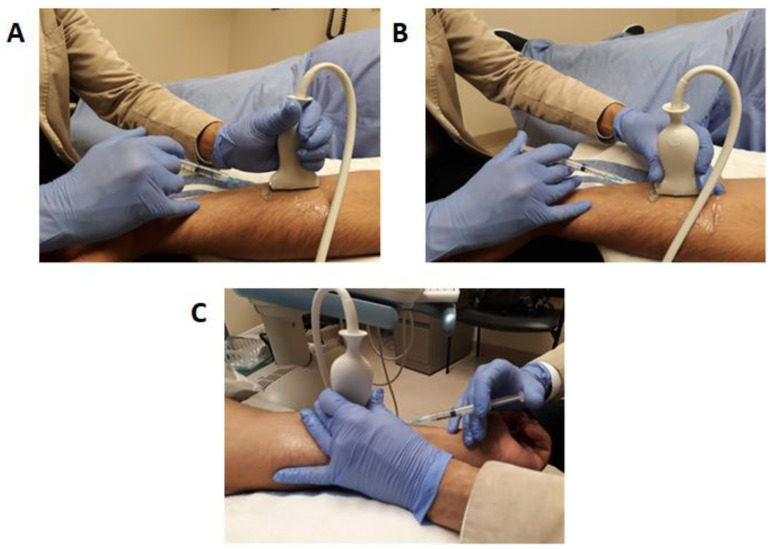
Transducer and needle grip. (**A**) Although the palmar grip reduces the required strength to apply a transducer on the skin, it may result in unintentional movement of the image during injection and makes millimetric shifting for toggling more complex. (**B**) An optimal probe grip with anchoring to the skin using the 4th and 5th digit increases stability. (**C**) Notice the injecting hand’s 5th digit is also used to stabilize while administering the product to reduce any undesired needle movement.

Transducer handling using non-dominant hand, the incorrect position of fingers on the transducer is associated with higher risks of injury and chronic pain [29,34]. An incorrect transducer grip for injection is shown in Figure 4A. We recommend handling the transducer using the index and middle finger with a contralateral thumb grip as this facilitates the use of the 4th and 5th digits to stabilize the ultrasound transducer against the patient’s skin as shown in Figure 4B,C. This prevents unintentional transducer movement during injection and facilitates easy rotation of the ultrasound transducer between the fingers while maintaining contact against the patient’s skin, allowing for a seamless transition from long-axis view to short-axis view while maintaining the area of focus on the ultrasound screen (Figure 4C).

Physicians should practice scanning and injecting ambidextrously as further practice can develop proficiency in both hands [35]. This is an important skill to develop as there may be situations where, due to room or patient factors, the set up does not allow for ergonomic positioning of the position for dominant hand injections.

### 4.4. Patient Ergonomics

Comfortable positioning of patients improves patient experience and safety by reducing the unintentional movement of the patient during the injection. In general, a comfortable position for a patient involves having the patient and the area injected well-supported by either the examination bed and pillows or by various other devices such as arm troughs and leg rest. If there are barriers to transferring a patient to a bed or the patient cannot tolerate a supine or prone position, the patient can be injected while sitting (in their wheelchair or another suitable chair). Additionally, for head/neck and some upper limb injections, a sitting position may provide better access to the region of interest.

Patients also often express interest in visualizing the ultrasound screen and procedure. Having a second screen and sharing the screen may be helpful by creating a positive placebo effect/biofeedback experience and is beneficial for the doctor-patient relationship. However, if one screen is used, it can result in a suboptimal position, resulting in the physician tilting his head or stretching his neck to share the monitor with the patient. Thus, having a second screen is considered optimal to facilitate patient involvement during the procedure (Figure 2A,C,D).

When injecting without assistance, the use of additional devices such as arm troughs or straps may be useful to maintain good exposure to the targeted muscle. However, when possible we recommend having an assistant available for the following reasons: physically assisting with the positioning of the patient for injection; providing emotional support to the patient during the procedure; monitoring the patient’s emotional reaction during injection as the physician may be focused on the area of injection and the ultrasound screen.

### 4.5. Visual Ergonomics Using Ultrasound

**Figure 5:**
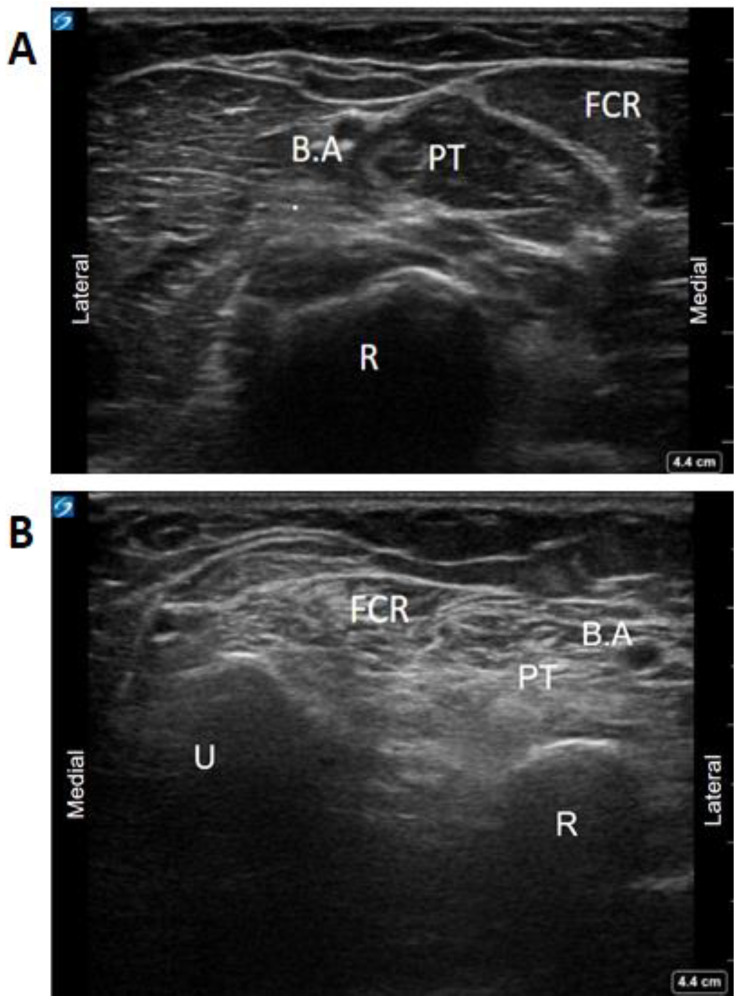
Comparison of normal and spastic muscle with fibrotic changes. (**A**) Unaffected Right forearm. (**B**) Affected left forearm with fibrotic changes. Architectural changes in spastic muscle with increased fibrosis on the affected limb modifying the appearance of fascial planes used to delineate the muscles. The use of bony landmarks and adequate scouting of the limb permits the recognition of muscle groups. (FCR: Flexor Carpi Radialis, PT: Pronator Teres, R: Radius, B.A: Brachial Artery, U: Ulna, R: Radius). [Images acquired on Sonosite Xport (FujiFilm Sonosite incorporated, 21,919 30th Dr. SE, Bothell, WA, USA) using 15–6 Hz linear array transducer].

Adequate field of view and depth are necessary to recognize bony landmarks. Patients who have had long-standing BoNT injections and increased spasticity often have increased muscle fibrosis leading to loss of muscle fascial planes used to delineate muscles from each other [12,36,37]. In these cases, it may be necessary to use bony landmarks such as the tibia and the interosseous membrane for identifying tibialis posterior or the radius and ulna for flexor compartment muscles as a strategy to identify fibrosed muscles under ultrasound (Figure 5) and use of the Modified Heckmatt Scale for identification of muscle fibrosis [36].

**Figure 6:**
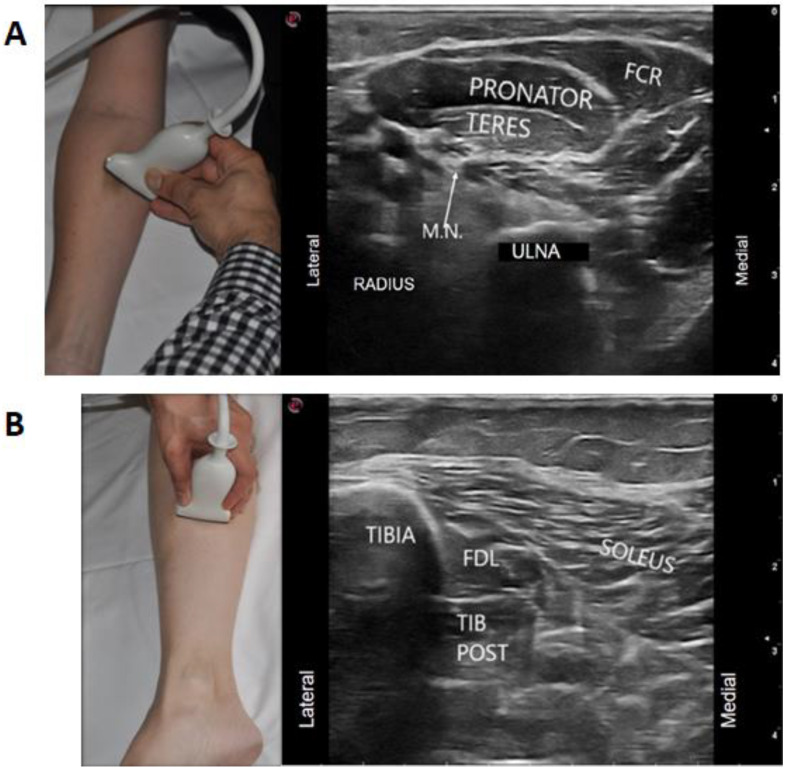
Visual ergonomics. (**A**) Positioning transducer one transversal length down from the cubital fossa offers a scouting landmark: FCR can be recognized as a “shark eating a ball”, in this case, the ball referring to pronator teres. (FCR: Flexor Carpi Radialis, PT: Pronator Teres, M.N.: Median Nerve). (**B**) Flexor digitorum longus having an appearance of a “sail” or “shark-fin” just lateral to the tibia can be used to identify other muscles such as the tibialis posterior separated by a fascial plane just underneath. (Images acquired on Esaote MyLab 7 ultrasound machine, Esaote ultrasound machine with 13–3 Hz linear array transducer, (Esaote; location: Fishers, IN, USA)).

**Figure 7:**
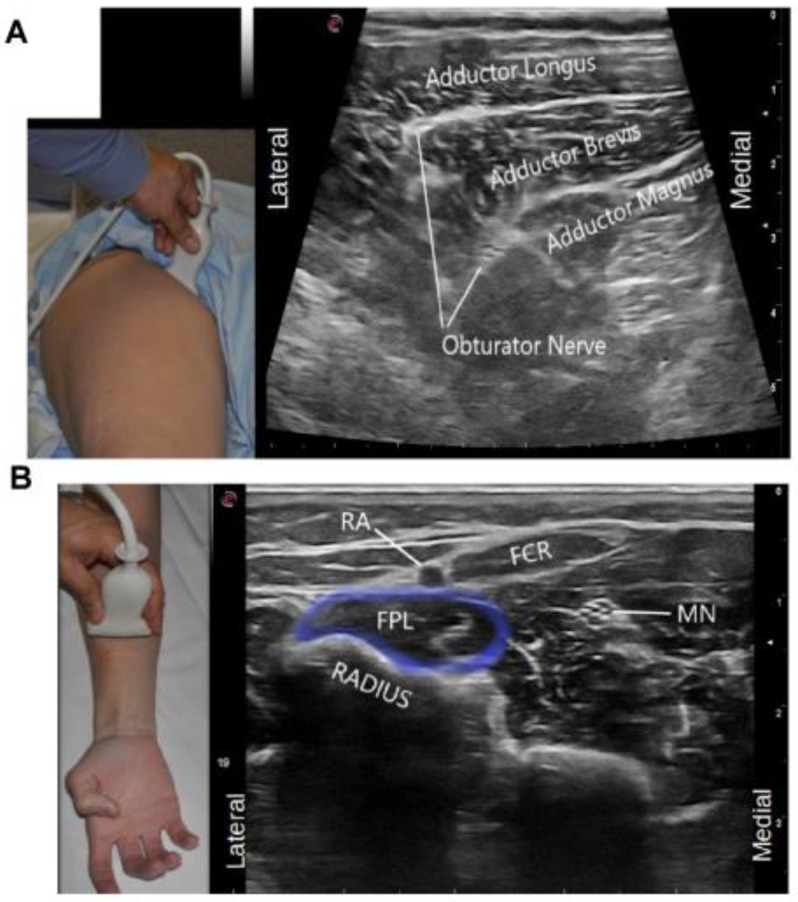
Visual Ergonomics. (**A**) Anterior and posterior branches of the obturator nerve can be used to delineate the fascial planes of the adductor longus, brevis and magnus (**B**) Visualisation of the median nerve can be used as landmark to identify the Flexor Pollicis Longus. Identification of these nerve to muscle relationships can also be key if nerve blocks are to be performed for diagnostic injection to determine contracture versus spasticity (FCR: Flexor Carpi Radialis, RA: Radial artery, MN: Median Nerve, FPL: Flexor Pollicis Longus). (Images acquired on Esaote MyLab 7 ultrasound machine, Esaote ultrasound machine with 13–3 Hz linear array transducer, (Esaote; location: Fishers, IN, USA)).

Appropriate selection of the ultrasound probe (depending on the depth of the muscle); focus zone selection on the ultrasound machine and adjustment of gain to optimize the image quality on the screen are also important. These were identified by 73% of our experts as a common ergonomic error among their trainees. This facilitates the use of pattern recognition which is a common strategy for muscle identification under ultrasound. For example, in the upper limb, shape recognition of the round muscle belly of the pronator teres muscle resembling a “ball” and flexor carpi radialis (FCR) looking like a “shark mouth” can be used by stating that the FCR looks “like a shark” eating the “the ball” of pronator teres (Figure 6A). Optimal image quality also allows for recognizing neurovascular structures that may also be helpful for muscle localization. An example is the localization of the median nerve to identify flexor digitorum superficialis two (FDS2), which is more medial to the median nerve when compared to flexor digitorum superficialis three (FDS 3) [38]. In the lower limb, the relationship of flexor digitorum longus having an appearance of “a sail” or “shark-fin” can also help in identifying and targeting this muscle (Figure 6B). Another example where localization of the neurovascular structures can be helpful in muscle localization is the use of the anterior and posterior branches of the obturator nerve to delineate the fascial planes of the adductor longus, brevis and magnus, as these muscles do not have clear fascial boundaries on ultrasound (Figure 7A).

Identification of these nerve-to-muscle relationships can also be important if diagnostic nerve blocks are used to distinguish contracture versus spasticity (Figure 7A,B).

Appropriate selection of acoustic windows will also help determine the best needle pathway/approach. For instance, there are three different approaches to inject tibialis posterior (dorsal, anteromedial, ventral), and depending on the patient’s age and position, the muscle depth and neuro-vascular bundle change, making certain approaches preferable.

Furthermore, proper use of gain and ultrasound probe selection can help determine the level of muscle echointensity (spastic muscles are more hyperechoic secondary to muscle atrophy, increased fat infiltration and fibrosis), which can be visually assessed by the Modified Heckmatt scale for spasticity [36]. Picelli et al. demonstrated that outcomes to BoNT may be affected if a spastic muscle is more fibrotic [37]. Furthermore, there is a study that hypothesizes that identification and injection of the toxin into hypoechoic pockets of muscle can theoretically enhance the uptake of BoNT [36].

Proper orientation of the ultrasound transducer is necessary for the desired medial/lateral, proximal/distal orientation. In radiographic convention, when scanning in cross-section, the transducer notch, line, or light is placed to orient left on the display screen. This results in images similar to CT or MRI images, where structures on the right side of the body (such as the liver) are displayed on the left side of the screen. This is referred to as “standard cross-sectional imaging” [31].

**Figure 8:**
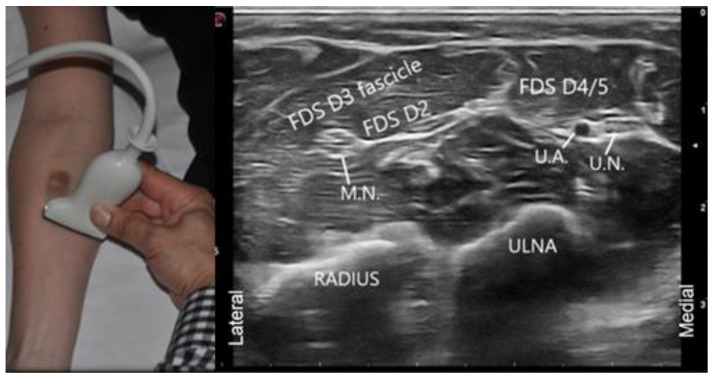
Notch positioning. When injecting the right upper limb, we suggest that the notch be placed laterally as opposed to the radiologic convention of notch medial. The position of the notch laterally allows for the ultrasound image to be a direct reflection of the movement of the ultrasound probe on the skin. (FDS: Flexor Digitorum Superficialis/Sublimis, M.N.: Median Nerve, U.A. Ulnar Artery, U.N.: Ulnar Nerve) (Images acquired on Esaote MyLab 7 ultrasound machine, Esaote ultrasound machine with 13–3 Hz linear array transducer, (Esaote; location: Fishers, IN, USA)).

This convention can work well if injecting a patient’s left limb with the ultrasound screen at the head of the patient, as the notch will be medial to the patient. However, when injecting the right limb with the ultrasound screen at the head of the patient, this convention can be counter intuitive. As can be seen by the figure below (Figure 8), if the notch or other transducer mark were positioned medially, as the transducer moves from the injector’s left to right (i.e., patient’s lateral to medial), the ultrasound image on the screen will move in an opposite direction (i.e., move from right to left). Therefore, it is more intuitive to position the ultrasound transducer notch laterally when injecting the right limb, as demonstrated in the picture. In this setup, as the transducer moves from the injector’s left to right (i.e., patient’s lateral to medial), the image on the ultrasound machine will also move from left to right, facilitating image interpretation during dynamic scanning.

Our expert panel agrees that the transducer notch, light, or line should be positioned such that movement of the ultrasound image is a direct reflection of the movement of the ultrasound probe on the skin (i.e., simplified cross-sectional imaging). Nonetheless, physicians must be familiar with standard cross-sectional imaging as well, as images produced in the scientific literature often follow this convention.

## 5. Conclusions

Ergonomics in US-guided BoNT chemodenervation for spasticity should be optimized to ensure the best outcomes for both the physician and the patient. It currently remains an overlooked aspect of US-guided chemodenervation education courses in spasticity management. With the emergence of more accessible ultrasound devices resulting in its increasing use, physicians should take the necessary time before procedures to plan their approaches and optimize positions, which may ultimately increase comfort for both physicians and patients. As ultrasound hardware technology improves, the equipment will become smaller in size and smarter (i.e., handheld tablet, Bluetooth technology) and will likely require regular adjustment to ergonomic standards. Improved ergonomics in US-guided BoNT chemodenervation could also potentially improve clinical outcomes by reducing the probability of unintentional probe movement and increase the accuracy of injection, but this requires further study. Furthermore, novice physicians integrating ultrasound into their practice could benefit from ergonomic education and this education should be covered during US-guided BoNT for spasticity courses. This would help raise awareness of best practices and could minimize and prevent injuries before “bad habits/reflexes” are acquired. Future articles exploring the long term economic and productivity outcomes of such interventions would help identify and reinforce best practices. We hope this article will be a useful resource for novice physicians in developing sound ergonomic practices in US-guided BoNT chemodenervation for spasticity. For more experienced physicians, we hope to produce future articles addressing more advanced ergonomic issues in US-guided chemodenervation such as choosing in-plane/out-of-plane injections, ambidextrous injections, and injecting spastic patients with complex patterns of spasticity resulting in abnormal positioning of the limbs. As the objective of this paper was to focus on ergonomics in US-guided BoNT chemodenervation for spasticity, future publications can explore the role of ergonomics in US-guided diagnostic nerve blocks, as similar challenges and considerations exist for this important procedure in spasticity management. It is also important that real world research be conducted to explore whether ergonomics can affect US-guided BoNT injection outcomes. This knowledge, in addition to the inclusion of collaborative research with ultrasound experts, will help in the development of guidelines for the use of ultrasound in BoNT chemodenervation.

## 6. Methods

As the current state of literature in Polymyalgia rheumatica (PMR) is insufficient to generate ergonomic guidelines, only strategies based on the opinion of experts can be offered. A working group of four individuals generated a list of 20 statements to form the basis of an initial survey (Supplementary Materials) to an international group of experts.

The international group of US chemodenervation experts consisted of 11 experienced physicians in the treatment of spasticity (10 PMR specialists, 1 neurologist) from North America, Europe and Asia with academic university appointments and involved in the development and teaching of US-guided injection courses at a national and international level in the USA, Canada, Italy, Switzerland, Germany and Thailand. An opinion was considered consensus if 80% agreement on a statement was obtained. However, in situations where 80% consensus opinion was not achieved, discussions were held with the experts concerning these areas of disagreements. Based on these discussions, the consensus opinion on the most common ergonomic mistakes seen by the experts when teaching was created.

## Figures and Tables

**Table 1:** Selected Survey Responses.

Question	Mean (Range)
How many years of experience do you have regarding the use of ultrasound-guided chemodenervation for spasticity management?	12.2 years (3–22)
How many years of teaching do you have with regards to training medical students, residents and other junior staff physicians in the area of ultrasound-guided chemodenervation?	10.5 years (2–20)
In the courses you have attended for ultrasound-guided injection, what percentage formally addressed the proper ergonomics of ultrasound-guided injections?	17.3% (0–50)
**Question & Responses**	**Responses (%)**
When you perform ultrasound-guided chemodenervation do you operate alone or with another person assisting?	
**Alone**	0 (0%)
**Assisted**	4 (36.4%)
**Both**	7 (63.6%)

## Data Availability

Not Applicable.

## Supplementary Materials

Sample Questionnaire are available online at https://www.mdpi.com/article/10.3390/toxins13040249/s1.

## Abbreviations

Botulinum neurotoxin	BoNT
Ultrasound	US
Work-Related Musculoskeletal Disorders	WRMSDs
Musculoskeletal	MSK
