# Supplementary material for: Ergonomic Recommendations in Ultrasound-Guided Botulinum Neurotoxin Chemodenervation for Spasticity: An International Expert Group Opinion
Source: Toxins (Basel). 2021 Mar 31;13(4):249. doi: 10.3390/toxins13040249 (PMC8067138; doi:10.3390/toxins13040249)
Supplement: Supplementary file 1 [file toxins-13-00249-s001.pdf]

Supplementary Materials: Ergonomic Recommendations in Ultrasound-Guided Botulinum Neurotoxin Chemodenervation for Spasticity: An International Expert Group Opinion

Philippe Lagnau, Alto L, Ryan Sandarag, Katharine Alter, Alessandro Picelli, Jorg Wissel, Monica Verduzco-Gutierrez, Areerat Suputtitada, Michael C. Munin, Stefano Carda, Omar Khan, Serdar Koçer and Rajiv Reebye

Sample Questionnaire

Ergonomics in Ultrasound-Guided Chemodenervation for Spasticity Management

Thank you for taking the time to complete the following survey. You have been identified as an expert for ultrasound-guided chemodenervation. We are using your responses to formulate consensus guidelines to improve ergonomics for ultrasound-guided chemodenervation. The following survey should take approximately 30 minutes to complete.

All images of the patient have been taken with their permission. Images shown are simulated to include both good and bad elements of ergonomics to elicit a range of responses.

What is your name?

Q1: How many years of experience do you have regarding the use of ultrasound-guided chemodenervation for spasticity management?

Q2: How many years of teaching do you have with regards to training medical students, residents and other junior staff physicians in the area of ultrasound guided chemodenervation?

Q3: In the courses you have attended for ultrasound guided injection, what percentage formally addressed the proper ergonomics of ultrasound guided injections?

Q4: In your opinion, why is optimizing ergonomics important? Choose all that apply:

- o Reduces risk of injury to the physician
- o Allows for clinics to run more efficiently
- o Reduces risk of adverse events to patients (such as infection, bleeding)
- o Improves patient outcomes post-injections

Q5: When you perform ultrasound-guided chemodenervation do you operate alone or with another person assisting?

- Alone
- Another person assisting
- Both (mix of solo and with an assistant)

Q6: If you answered "Both" in the previous question, what would you do differently as a solo operator versus with a person assisting in regard to ergonomics?

Q7: Which of the following, do you consider essential in optimizing the ergonomics for an ultrasound guided injection for spasticity management? You may choose multiple options.

- Physical setup of the room (including use of multiple displays, cord management)
- Patient Positioning
- Physician Positioning
- Visual targeting (pattern recognition, bony, vascular or nerve landmarks)
- Positioning of the procedure tray
- Positioning of assistant (if used)
- Positioning of the Ultrasound Machine
- Add option

Q8: Do you feel that proper ergonomics have a positive functional impact on post-injection outcomes?

- Yes
- No
- Maybe

Injection of extensor hallucis longus

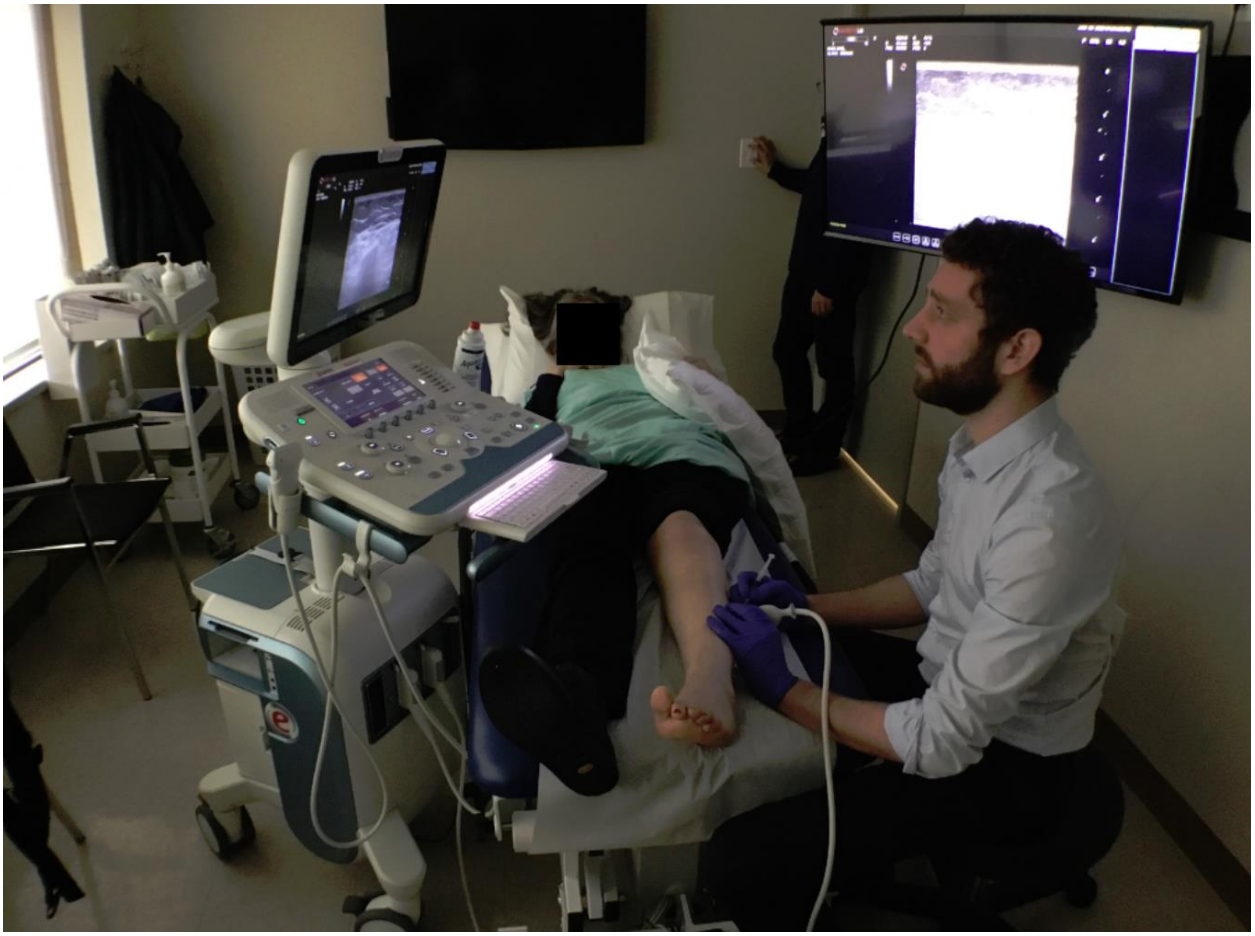

Q9: In the above image, please list 3–5 POSITIVE ergonomic elements? If you have more points, please feel free to add more if needed.

Q10: In the above image, please list 3–5 things you would change to IMPROVE the ergonomics of this injection? If you have more points, please feel free to add more if needed.

Injection of biceps brachii

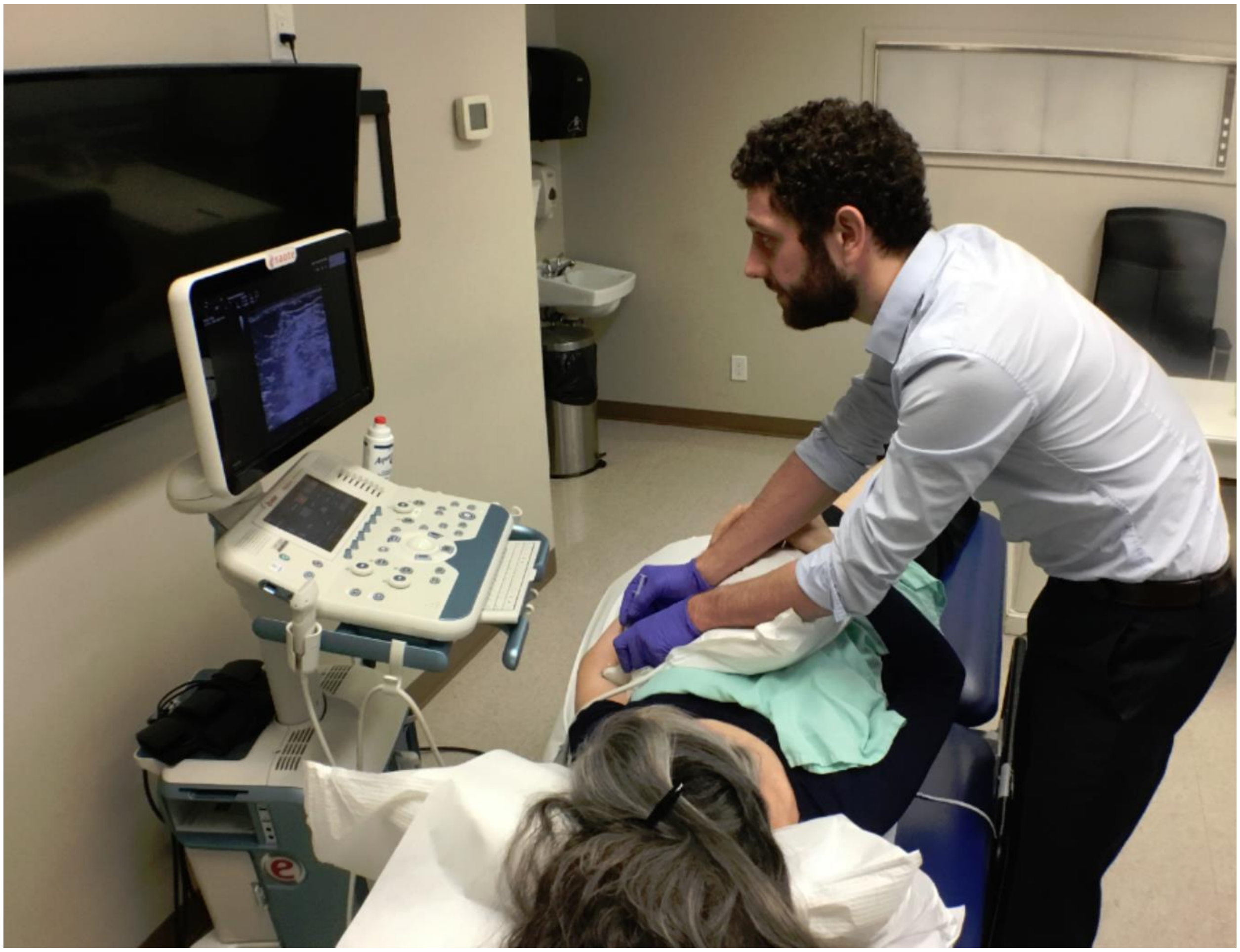

Q11: In the above image, please list 3–5 things you would change to IMPROVE the ergonomics of this injection? If you have more points, please feel free to add more if needed.

Injection of medial gastrocnemius

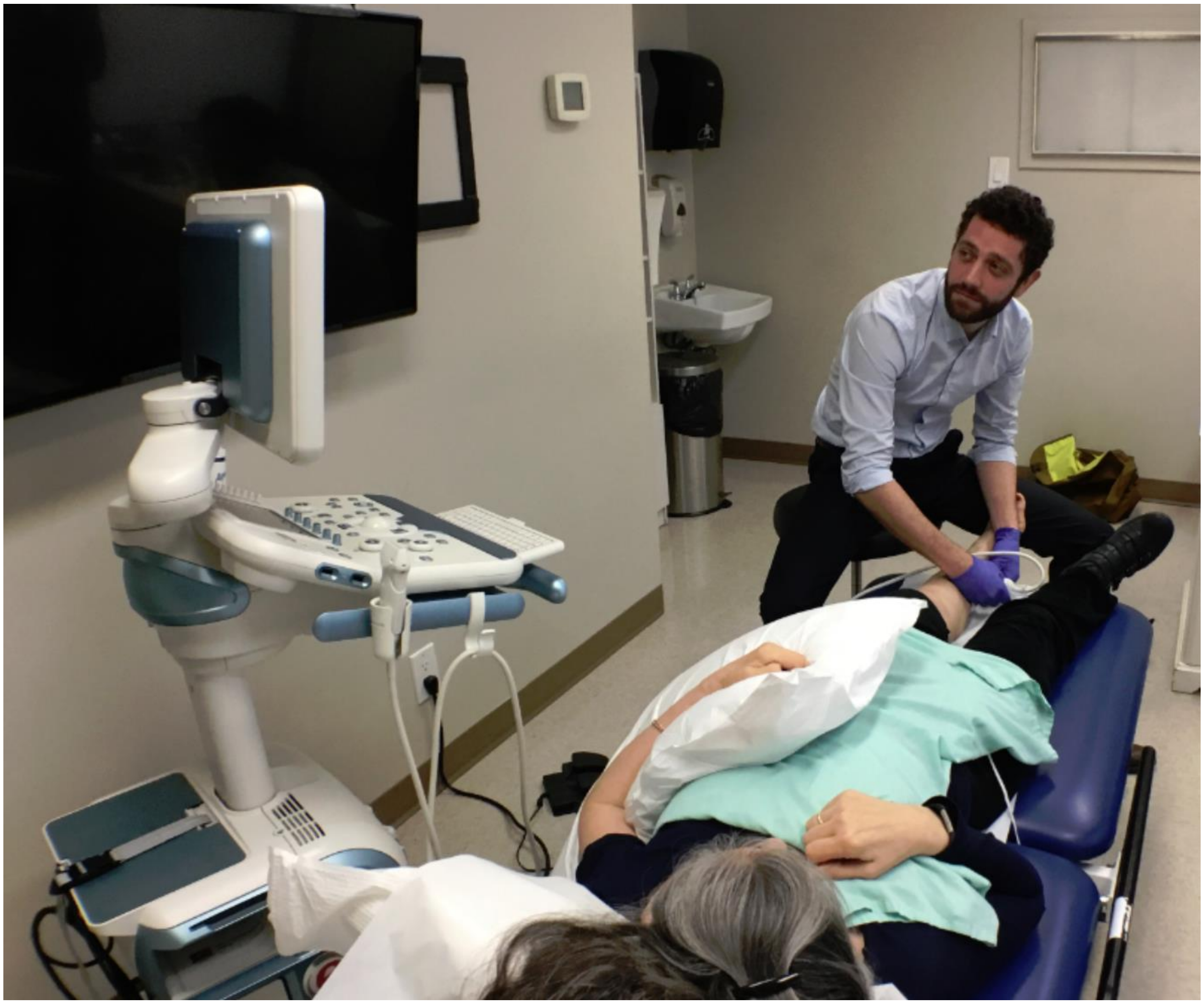

Q12: In the above image, please list 3–5 things you would change to IMPROVE the ergonomics of this injection? If you have more points, please feel free to add more if needed.

Injection of brachioradalis

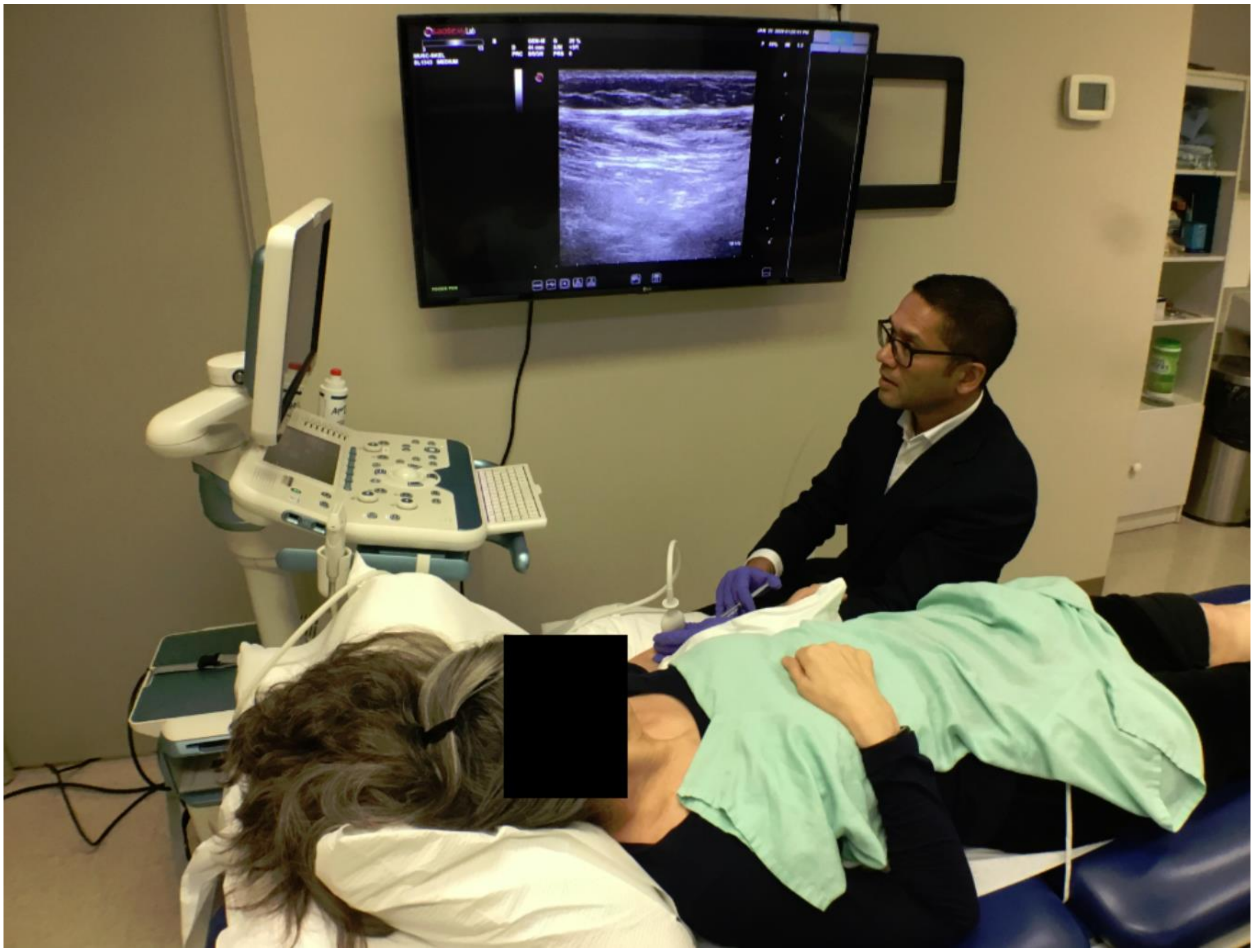

Q13: In the above image, please list 3-5 POSITIVE ergonomic elements? If you have more points, please feel free to add more if needed.

Q14: In the above image, please list 3-5 things you would change to IMPROVE the ergonomics of this injection? If you have more points, please feel free to add more if needed.

Scenario 1: Ultrasound probe and needle positioning

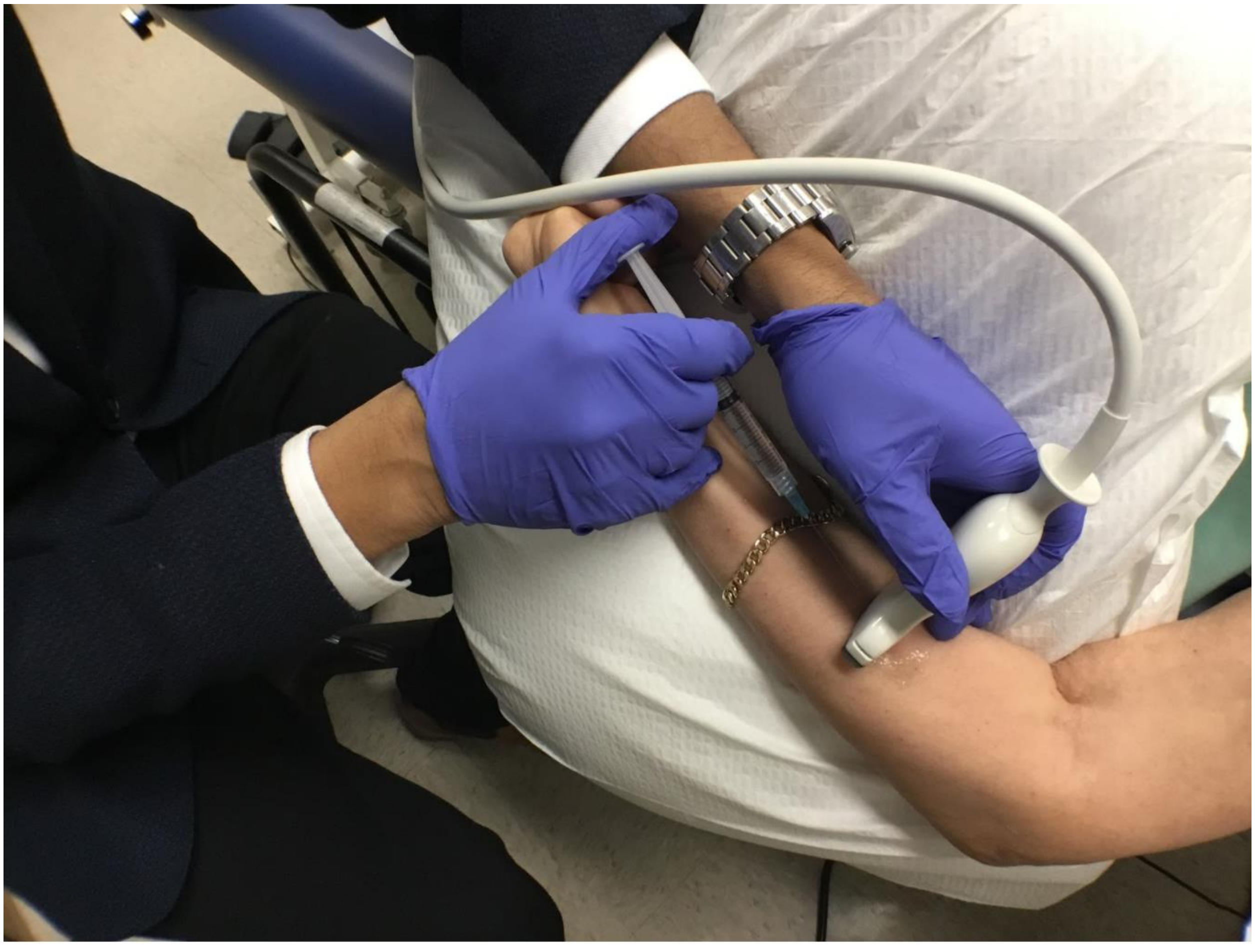

Scenario 2: Ultrasound probe and needle positioning

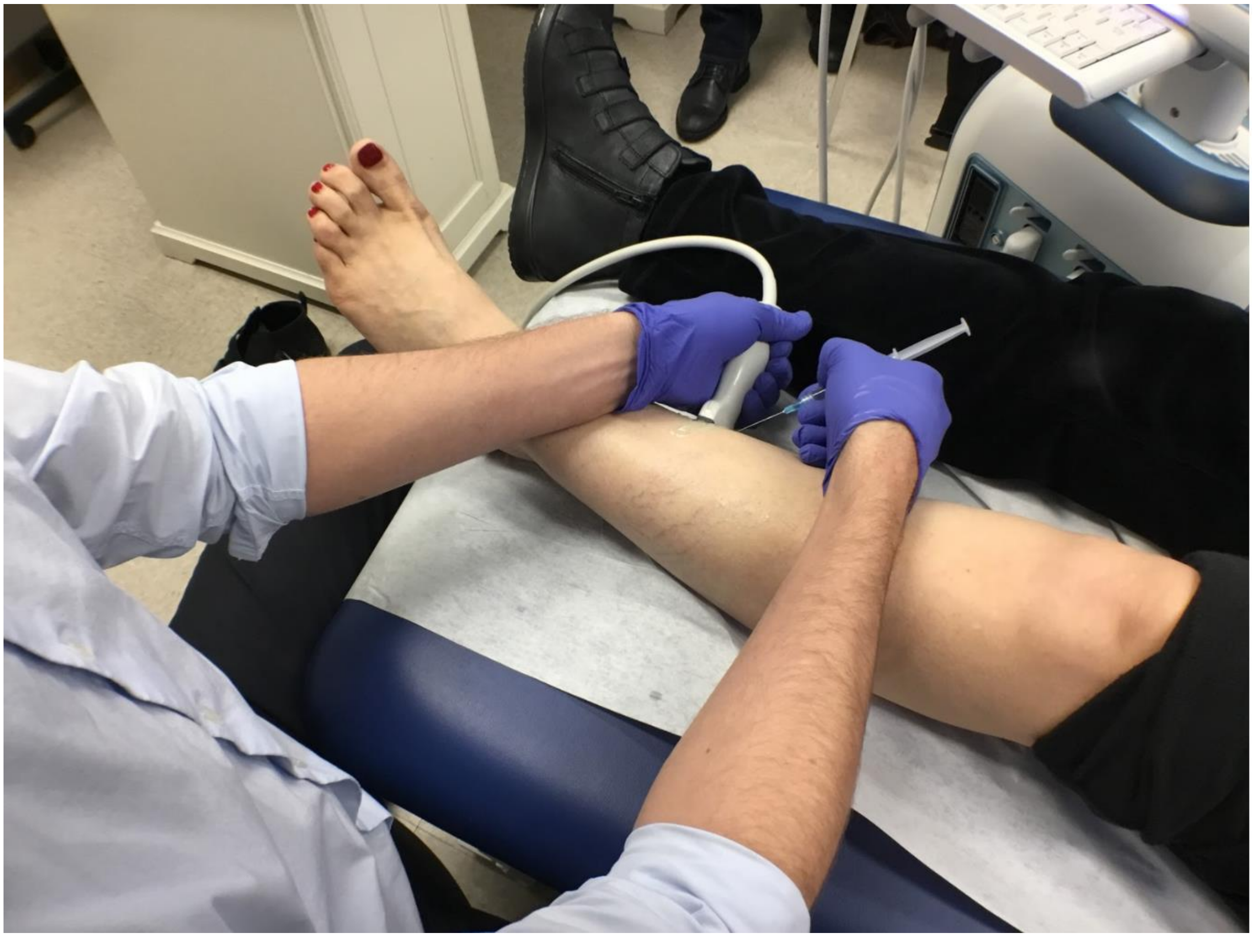

Q15: Comparing scenarios 1 and 2, which scenario would you consider being preferable positioning of transducer and needle?

- Scenario 1
- Scenario 2

Q16: In the scenario that you consider preferable, list 3 to 5 elements that have been improved to optimize ergonomic positioning of transducer and needle?

Scenario 1: Patient & physician positioning

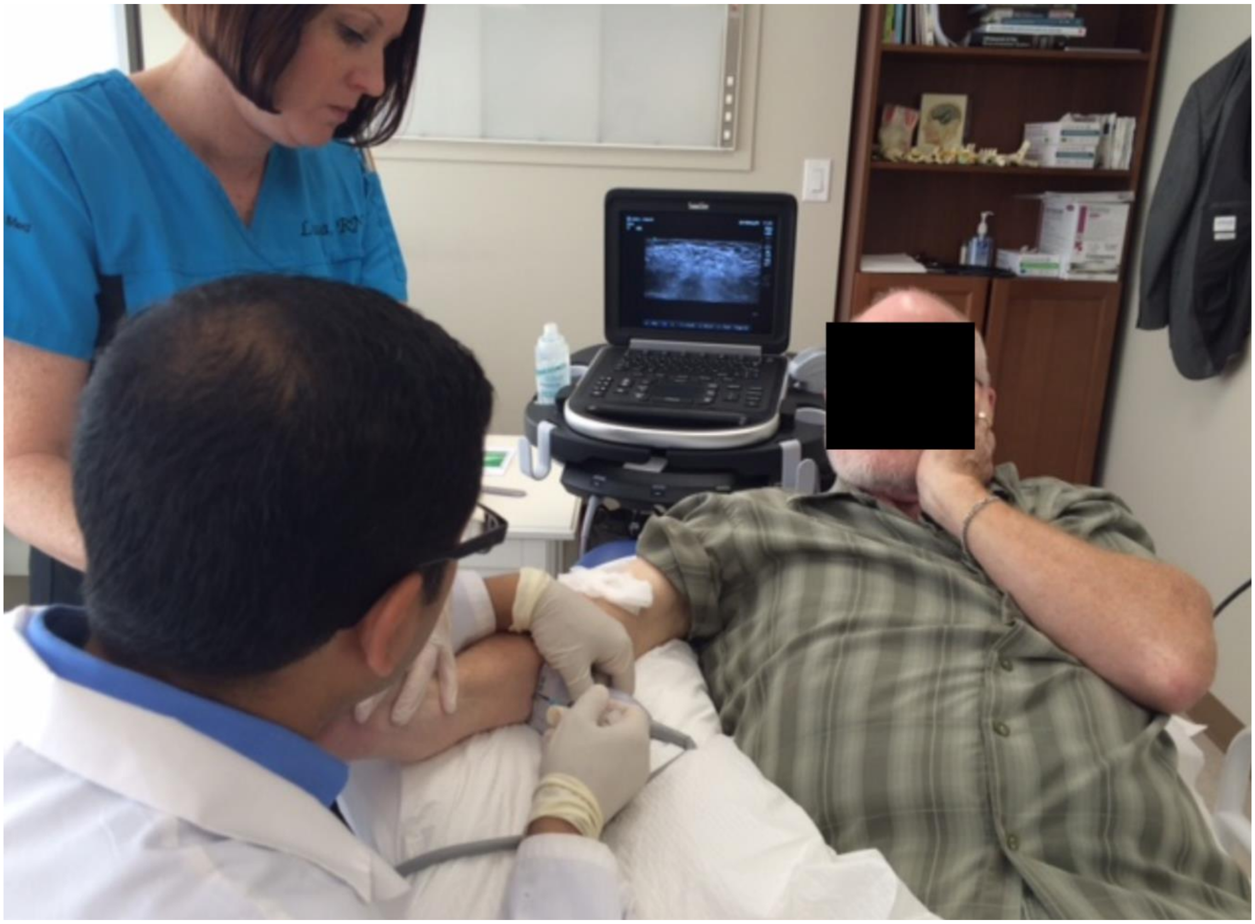

Scenario 2: Patient & physician positioning

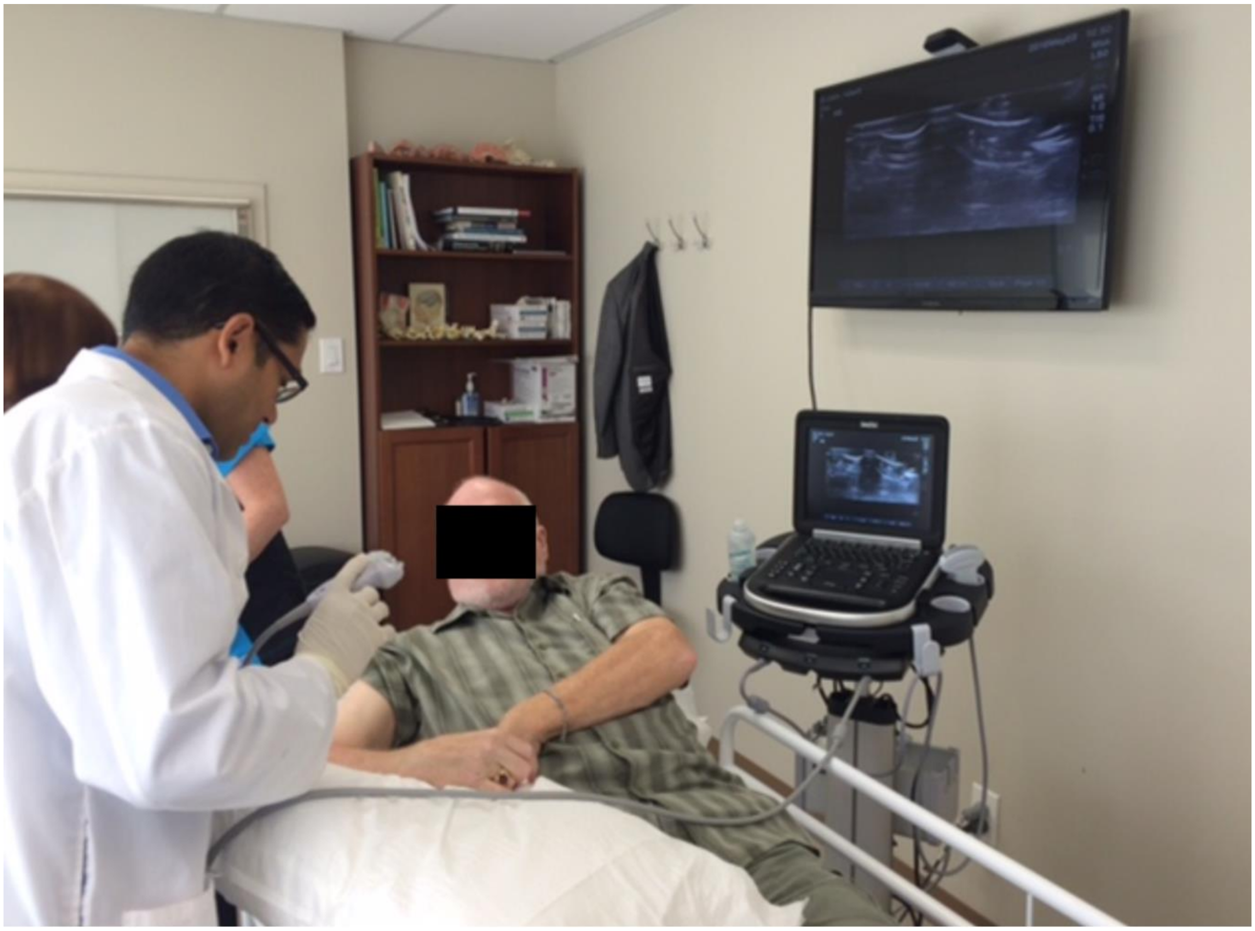

Q17: Comparing scenarios 1 and 2, which scenario would you consider being preferable positioning?

- Scenario 1
- Scenario 2

Q18: In the scenario that you consider preferable, list 3 to 5 ergonomic elements that have been improved to optimize ergonomic positioning of the patient, physician, and ultrasound?

Q19: When presented with the following image, which visual ergonomic strategies do you apply when identifying and injecting Flexor Carpi Radialis ?

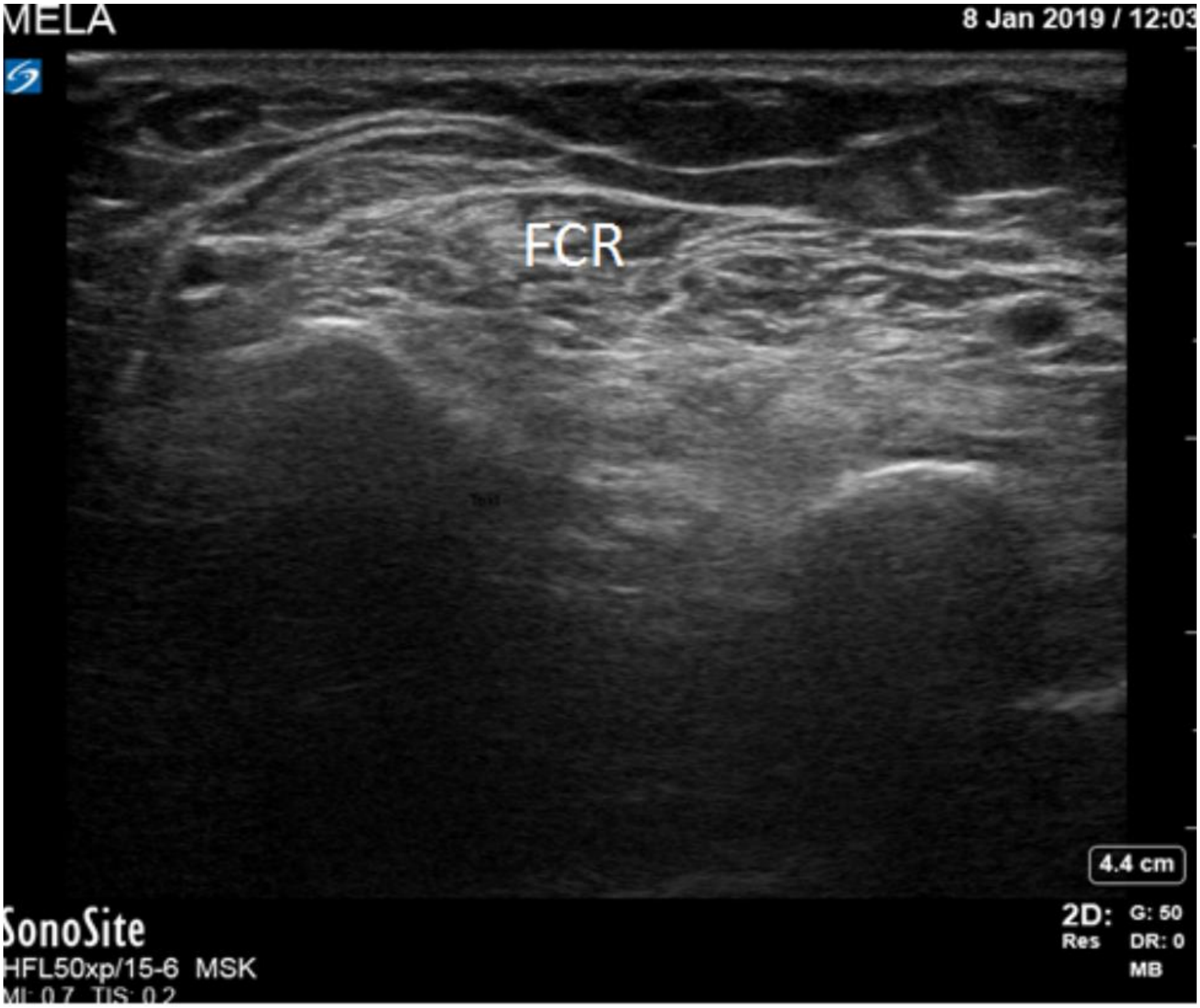

- Pattern recognition is important in identification of key muscles to inject
- Bony landmarks are key in assessment of location of key muscles to inject
- Muscle hypo or hyper-echoic muscle regions are important to assess
- Neuro-vascular structures are important to visualize to identify muscles to inject
- Add option

Q20: Below find a list of ergonomic mistakes seen when teaching medical students/residents. Please select the ergonomic mistakes you commonly see in your teaching practice. You may select as many as you wish:

- Insufficient transducer gel
- Inadequate grip of probe/transducer
- Poor posture during injection
- Improper positioning of the US machine so that the controls are out of reach

- Improper positioning of the procedure tray and other necessary equipment because they are out of reach
- Inadequate visualization/scouting of nearby structures to identify potential areas of hazard and for landmarking purposes
- Inadequate image optimization through adjustment of focus, depth and transducer frequency leading to poor visualization of target
- Poor needle path planning resulting in multiple structures pierced
- Improper placement of the ultrasound screen resulting in excessive movements when changing focus of vision from ultrasound screen to patient's skin during injection
- Improper positioning of the upper extremity (arm, wrist and fingers) that may lead to overuse injuries or fatigue
- Add option
